# Enhancing the methanol tolerance of platinum nanoparticles for the cathode reaction of direct methanol fuel cells through a geometric design

**DOI:** 10.1038/srep16219

**Published:** 2015-11-18

**Authors:** Yan Feng, Feng Ye, Hui Liu, Jun Yang

**Affiliations:** 1 State Key Laboratory of Multiphase Complex Systems, Institute of Process Engineering, Chinese Academy of Sciences, Beijing, China 100190; 2 University of Chinese Academy of Sciences, No. 19A Yuquan Road, Beijing, China 100190; 3 Center for Mesoscience, Institute of Process Engineering, Chinese Academy of Sciences, Beijing, 100190, China

## Abstract

Mastery over the structure of nanoparticles might be an effective way to enhance their performance for a given application. Herein we demonstrate the design of cage-bell nanostructures to enhance the methanol tolerance of platinum (Pt) nanoparticles while remaining their catalytic activity for oxygen reduction reaction. This strategy starts with the synthesis of core-shell-shell nanoparticles with Pt and silver (Ag) residing respectively in the core and inner shell regions, which are then agitated with saturated sodium chloride (NaCl) solution to eliminate the Ag component from the inner shell region, leading to the formation of bimetallic nanoparticles with a cage-bell structure, defined as a movable Pt core enclosed by a metal shell with nano-channels, which exhibit superior methanol-tolerant property in catalyzing oxygen reduction reaction due to the different diffusion behaviour of methanol and oxygen in the porous metal shell of cage-bell structured nanoparticles. In particular, the use of remarkably inexpensive chemical agent (NaCl) to promote the formation of cage-bell structured particles containing a wide spectrum of metal shells highlights its engineering merit to produce highly selective electrocatalysts on a large scale for the cathode reaction of direct methanol fuel cells.

Among various types of fuel cells, the direct methanol fuel cells (DMFCs) are most promising as the power sources for the portable and mobile products requiring low power density but high energy density. Their ability to operate at relatively low temperatures and quick start-up characteristics (since methanol is used directly without the need for fuel reforming) compares favourably with the polymer electrolyte membrane fuel cells (PEMFCs) based on hydrogen oxidation^1^,^2^,^3^. To recap briefly, one of the major problems with DMFCs after close to two decades of research efforts is the crossover of methanol from the anode to the cathode through the polymer electrolyte membrane (PEM), which can lead to a significant reduction of the fuel cell efficiency because the commonly used platinum (Pt) electrocatalyst at the cathode is not selective for oxygen reduction reaction (ORR), and it is also catalytically active for the methanol oxidation reaction (MOR)^4^,^5^,^6^,^7^,^8^. Although a number of efforts have been devoted toward the modification on the PEMs to overcome this key obstacle for the commercialization of DMFCs, it is generally thought that the commonly used Nafion membrane has an unacceptably high rate of methanol crossover^9^,^10^,^11^,^12^,^13^,^14^,^15^. In this sense, synthesis of electrocatalysts with high selectivity for ORR represents an alternative for solving the methanol crossover problem in DMFCs. Unfortunately, highly ORR selective electrocatalysts such as transition metal macrocyclic complexes^16^,^17^,^18^,^19^, transition metal sulfides and selenides^20^,^21^,^22^, are not chemically stable in the acidic environment of DFMCs and their low intrinsic ORR activities in the absence of methanol are known deficiencies. Therefore, the interest to develop methanol-tolerant cathode catalysts with ORR activities comparable to that of Pt has not waned over the years.

Instead of classical strategies for increasing the Pt catalytic performance through alloying with transition metals^23^,^24^,^25^,^26^, we advanced a concept in our early studies that the good ORR selectivity of the Pt catalyst could be realized through a geometric design, rather than making use of the intrinsic properties of the catalytic metal^27^,^28^. In this strategy, the bimetallic Pt-Ru nanoparticles with a cage-bell structure (CBS), which refers to a movable core enclosed by a shell with nano-channels, were produced based on the inside-out diffusion of Ag in Ag-containing core-shell metal nanoparticles to achieve the ORR selectivity. In the CBS Pt-Ru nanoparticles, the catalytically active metal, i.e. Pt, was located in the core region shielded by a porous Ru shell, which is inactive for methanol oxidation. The methanol and oxygen must diffuse into the CBS interior of the nanoparticle through the porous Ru shell for MOR and ORR to occur. However, a methanol molecule is larger than an oxygen molecule (the diameters of methanol and oxygen molecules are 0.44 nm and 0.34 nm, respectively). Hence the diffusion of O_2_ is faster than that of methanol in CBS Pt-Ru nanoparticles, rendering the oxidation of methanol on CBS Pt-Ru a non-competitive event.

Considering the great potential of CBS nanoparticles in catalysis^29^,^30^, we further attempt developing a more facile approach to the fabrication of CBS nanoparticles with active Pt residing in the core region based on the inside-out diffusion of Ag in Ag-containing core-shell metal nanoparticles. The research progresses in this work involve the optimization of Pt seed synthesis, the searching of a more cost-effective reagent as a substitute for the expensive bis(*p*-sulfonatophenyl)phenylphosphane (BSPP) used in previous studies to promote the inside-out diffusion of Ag, and the exploration of a wider spectrum of noble metals as the porous shell in CBS nanoparticles. As we will display later, the CBS nanoparticles show superior activity, stability, and selectivity for the ORR in the presence of high concentration of methanol. In addition, regarding the use of remarkably inexpensive chemical agent to promote the formation of CBS particles, the study in this work might have provided a promising method for creating highly selective electrocatalysts on a large scale for the cathode reaction of DMFCs.

## Results and Discussion

**Figure 1:**
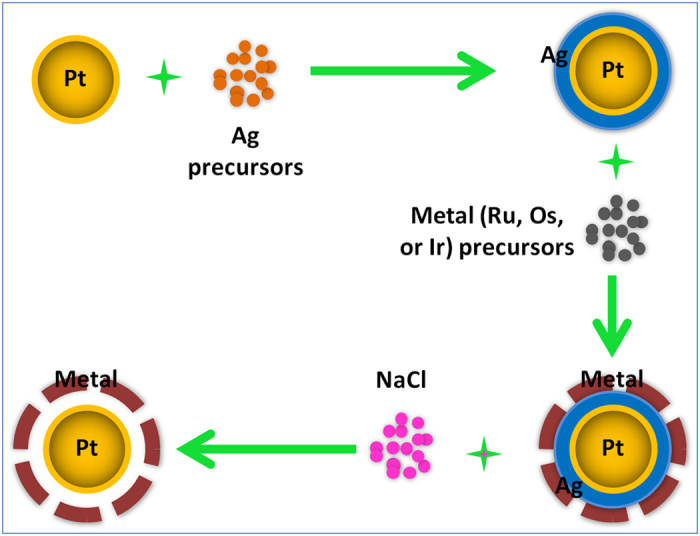
Synthetic strategy. Schematic illustration to show the synthesis of bimetallic Pt-M (M = Ru, Os, or Ir) nanoparticles with a cage bell structure.

Figure 1 shows the schematic illustration for the synthesis of bimetallic Pt-M (M = Ru, Os, or Ir) nanoparticles with a cage-bell structure. Analogous to that we reported earlier^27^,^28^, this protocol also begins with the synthesis of Pt seed particles in an organic solvent, which are overlaid with Ag shell first, followed by the growth of another metal (Ru, Os, or Ir) shell to form Pt-Ag-M nanoparticles with the requisite core-shell-shell structure. The inner Ag shell is then removed by saturated aqueous NaCl solution, which is much cheaper and easily obtained in comparison with the originally used bis(*p*-sulfonatophenyl)phenylphosphane (BSPP), leaving behind an organosol of CBS Pt-M nanomaterials.

**Figure 2:**
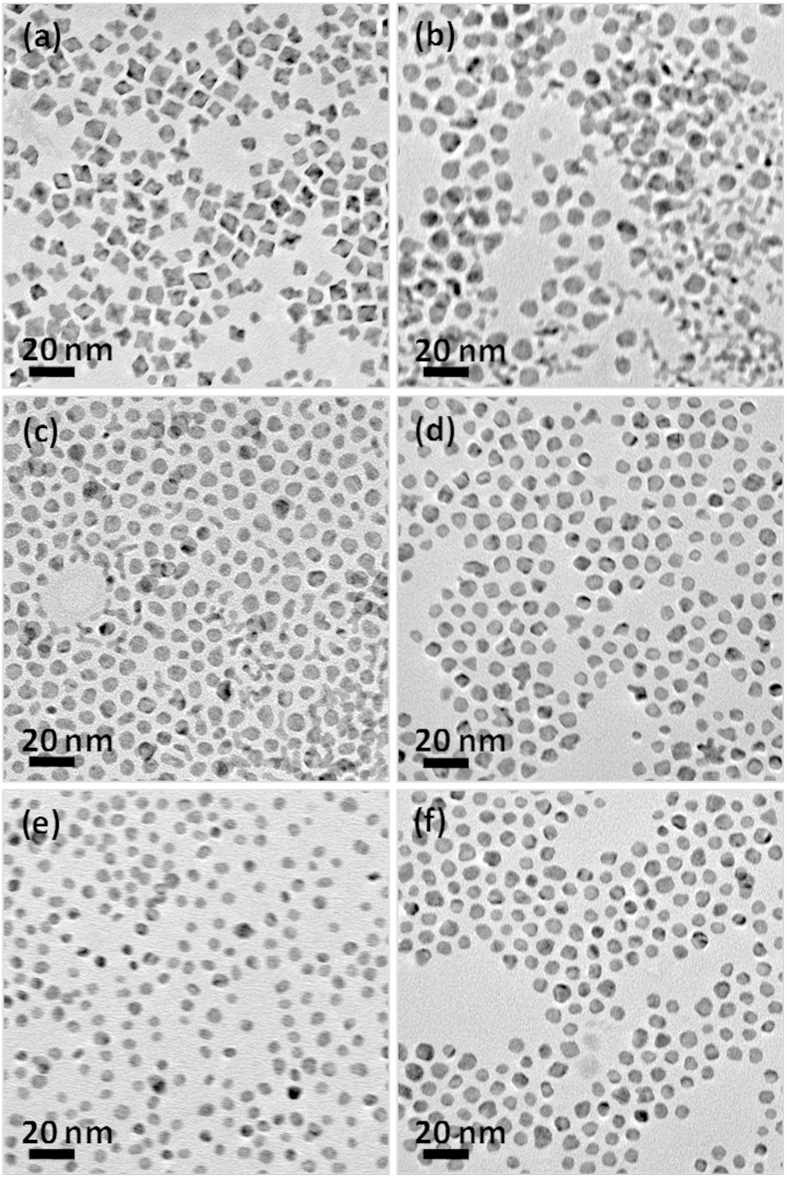
Platinum seed particles. Representative TEM images of the Pt seed particles synthesized at 155 °C (**a**), 160 °C (**b**), 170 °C (**c**), 180 °C (**d**), 185 °C (**e**), and 190 °C (**f**), respectively.

Figure 2 shows the transmission electron microscopy (TEM) images of the as-prepared Pt nanoparticles at different temperatures, which are used as seeds in the successive reduction reactions. As indicated, the reaction temperature has significant effect on the average size and morphology of the Pt seeds. At relatively low temperature (155°), the Pt nanoparticles are mostly polyhedral and have an average diameter of ca. 8.8 nm (Fig. 2a), while at relatively high temperatures (160 °C and 170 °C), the Pt nanoparticles thus obtained are quasi-spherical accompanied by a small portion of worm-like particles (Fig. 2b,c, respectively). When the synthesis temperature was increased to 180 °C, the Pt nanoparticles remain quasi-spherical morphology and almost have same size as that prepared at 160 °C and 170 °C, but the worm-like particles are not detectable at this temperature (Fig. 2d). At 185 °C, the Pt seeds are nearly spherical particles with an average diameter of ca. 4.5 nm (Fig. 2e). No apparent changes are observed in the particle size, morphology at higher reaction temperature, as evinced by Fig. 2f for the TEM image of the Pt particles synthesized at 190 °C.

As the seed particles with irregular morphologies would bring additional difficulty in latter formation of core-shell structures due to the reactivity of the different sites on their surface might be different^31^,^31^,^32^,^33^,^34^,^35^,^36^, the Pt nanoparticles synthesized at 185°C were chosen as the seeds for the subsequent reduction reactions. It should be mentioned that the addition of a small amount of AgNO_3_ was used to obtain Pt seed particles with regular spheres, without which the Pt nanotetrapods were the dominant products instead^27^,^37^. The influence of the additive amount of AgNO_3_ on the size/morphology of the Pt seeds was shown by the TEM images in Figure S1 of Supplementary Information (SI). As exhibited, the low AgNO_3_ addition cannot facilitate the formation of Pt spherical seeds, while the high additive AgNO_3_ would significantly increase the size of the Pt nanoparticles. In addition, as displayed by SI Figure S1d for the TEM image, when K_2_PtCl_4_ was used instead of Pt(acac)_2_ as Pt precursors, the Pt nanoparticles as-prepared have a stellated morphology rather than regular spherical shape, as has been reported in a recent literature^38^.

**Figure 3:**
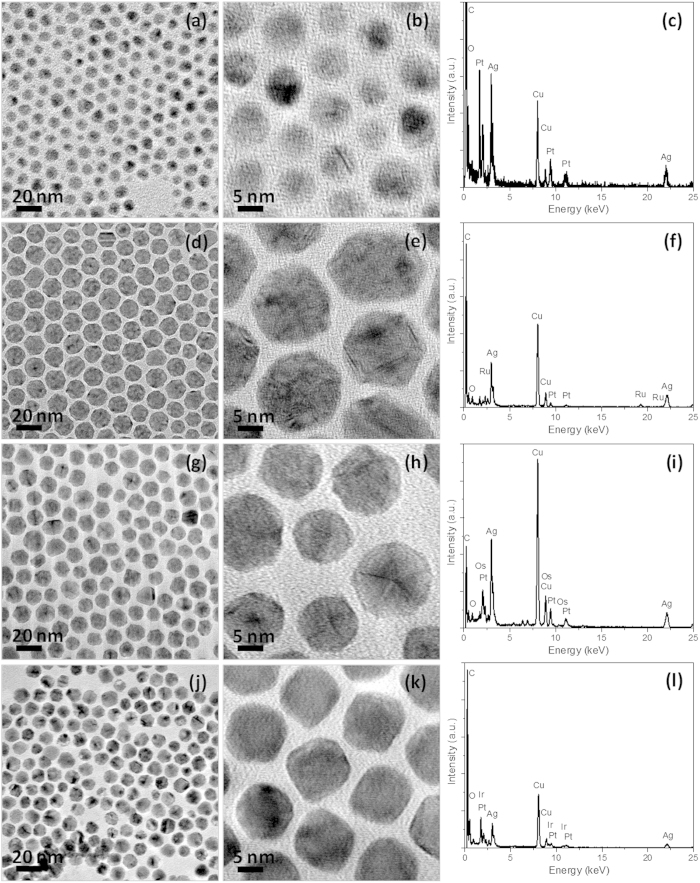
Core-shell-shell Pt-Ag-M nanoparticles. Representative TEM images (**a,d,g,j**), HRTEM images (**b,e,h,k**), and corresponding EDX spectra (**c,f,i,l**) of the as-prepared core-shell Pt-Ag (**a–c**), core-shell-shell Pt-Ag-Ru (**d–f**), core-shell-shell Pt-Ag-Os (**g–i**), and core-shell-shell Pt-Ag-Ir nanoparticles (**j–l**), respectively.

The preparation of core-shell Pt-Ag and core-shell-shell Pt-Ag-M (Ru, Os, or Ir) nanoparticles are the important steps preceding the preparation of CBS Pt-M nanostructures. Figure 3 shows the TEM and high-solution TEM (HRTEM) images of the core-shell Pt-Ag and core-shell-shell Pt-Ag-M nanoparticles presented in this study. Figure 3a,b are the typical TEM and HRTEM images of core-shell Pt-Ag nanoparticles, where the core-shell structure could be discerned by distinct brightness differences between the inner and outer regions of the particles. After overlaying with an Ag shell, the average diameter of the Pt seed particles increases from ca. 4.5 nm to ca. 8.6 nm. Upon reduction of Ru, Os, or Ir precursors in the presence of core-shell Pt-Ag nanoparticles, the average diameters of the final core-shell-shell Pt-Ag-Ru, Pt-Ag-Os, and Pt-Ag-Ir products reach 14.2 nm, 13.4 nm, and 10.2 nm, respectively, as demonstrated by the TEM (Fig. 3d,g,j, respectively) and HRTEM images (Fig. 3e,h,k, respectively). The existence of related elements in these core-shell products are confirmed by the corresponding EDX analyses (Fig. 3c,f,i,l for core-shell Pt-Ag, core-shell-shell Pt-Ag-Ru, Pt-Ag-Os, and Pt-Ag-Ir, respectively).

**Figure 4:**
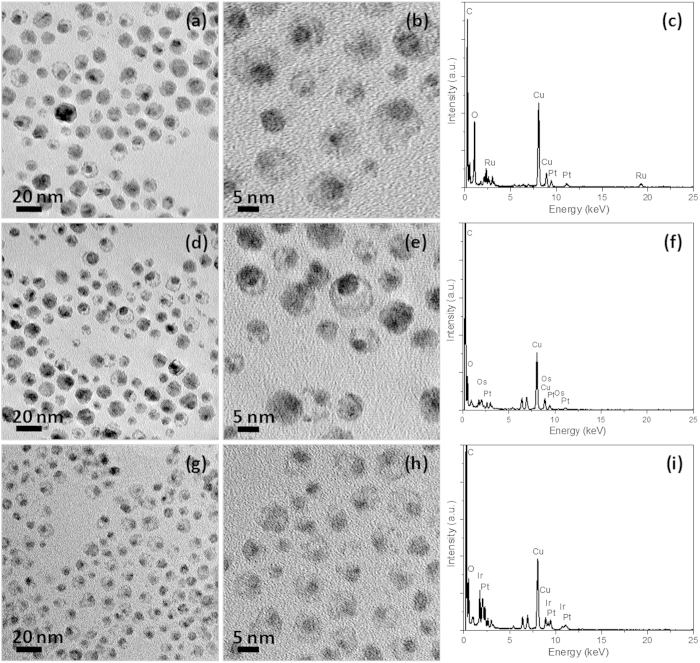
Cage-bell structured Pt-M nanoparticles. Representative TEM images (**a,d,g**), HRTEM images (**b,e,h**), and corresponding EDX spectra (**c,f,i**) of as-prepared CBS Pt-Ru (**a–c**), Pt-Os (**d–f**), and Pt-Ir nanoparticles (**g–i**), respectively.

After aging the toluene solution of core-shell-shell Pt-Ag-M nanoparticles with saturated aqueous NaCl solution for 24 h under vigorous stirring, the inner Ag layer is removed from the core-shell-shell Pt-Ag-M nanoparticles, leaving behind bimetallic Pt-Ru, Pt-Os, and Pt-Ir nanoparticles with the cage bell structures. SI Figure S2 shows the UV-visible spectra of the core-shell-shell Pt-Ag-M colloidal solutions before and after NaCl treatment, where the obliteration of the surface plasmon resonance (SPR), which is associated with the inner Ag layer as the other metals (Pt, Ru, Os, and Ir) do not have any absorbance, after the NaCl treatment might be used as an indirect evidence for the elimination of the inner Ag shell from the core-shell-shell Pt-Ag-M nanoparticles. More direct evidence was offered by the disappearance of Ag signal in the energy dispersive X-ray (EDX) spectra of core-shell-shell Pt-Ag-Ru, Pt-Ag-Os, and Pt-Ag-Ir nanoparticles after the NaCl treatment, as indicated by Fig. 4c,f,i, respectively. Electron microscopy images of the as-prepared CBS Pt-M nanoparticles are also given in Fig. 4, in which the void space between the core and the outer shell regions formed upon the elimination of the Ag inner shell by NaCl, is discernible by the strong brightness contrast in TEM and HRTEM images. As observed, in some cases, the entrapped cores are adhered to the inner surfaces of the outer shell due to the movable nature of the core after removal of the inner shell restraint. A comparison between the electron microscopy images of CBS nanoparicles (Fig. 4) and their core-shell-shell counterparts (Fig. 3) indicates that the particle size and morphology were virtually unchanged after the NaCl treatment, manifesting that the removal of the inner Ag shell from the core-shell-shell nanoparticles does not cause the collapse of the particle geometry. Further, the successful elimination of the inner Ag shell from the core-shell-shell Pt-Ag-M nanoparticles suggests the presence of tiny channels in the outer metal layer composed of Ru, Os, or Ir, which are not only the basis for the removal of the Ag component by NaCl, but also the prerequisite for the CBS nanoparticles to have electrocatalytic activities as the active Pt metal is located at the core region.

X-ray photoelectron spectroscopy (XPS) was used to analyze the chemical states of the elements in CBS Pt-M nanoparticles recovered from toluene. SI Figure S3a,c, and e show the 4f regions of Pt in CBS Pt-Ru, Pt-Os, and Pt-Ir nanoparticles, while the Ru 3p, Os 4f, and Ir 4f regions are given as SI Figure S3b,d,f, respectively. In Pt, Ru, and Ir, all spectra can be deconvoluted into two pairs of doublets. The more intense doublet (at 71.5 and 74.8 eV for Pt 4f, at 462.5 and 485.0 eV for Ru 3p, and at 61.4 and 64.4 eV for Ir 4f, respectively) is a signature of metals at zero valent state^39^,^40^. The second and weaker doublet (at 72.9 and 76.2 eV for Pt 4f, at 464.3 and 486.8 eV for Ru 3p, and at 62.4 and 65.7 eV for Ir 4f, respectively), with binding energies higher than those of zero valent metals, could be assigned to the oxidation state of the metals corresponding to PtO or Pt(OH)_2_, RuO_2_, and IrO_2_^39^,^40^. For Os in CBS Pt-Os nanoparticles, besides the metal at zero valent state, the oxidized states corresponding to Os^4+^(OsO_2_) and Os^8+^(OsO_4_) are also observed^40^, as shown in SI Figure S3d.

Both the CBS Pt-M nanoparticles and the Pt seeds were loaded on Vulcan carbon (labelled as CBS Pt-Ru/C, CBS Pt-Os/C, CBS Pt-Ir/C, and Pt/C, respectively) and tested for electrocatalytic activity for the ORR and MOR at room temperature. As shown by the representative TEM and HRTEM images in SI Figure S4, the Pt seeds and CBS P-M nanoparticles could be dispersed very well on the carbon support by conventional means and the cage-bell structure of the nanoparticles is intact. The loading of the Pt seeds and CBS particles on carbon was fixed at 20 wt% of Pt in order to be comparable.

**Figure 5:**
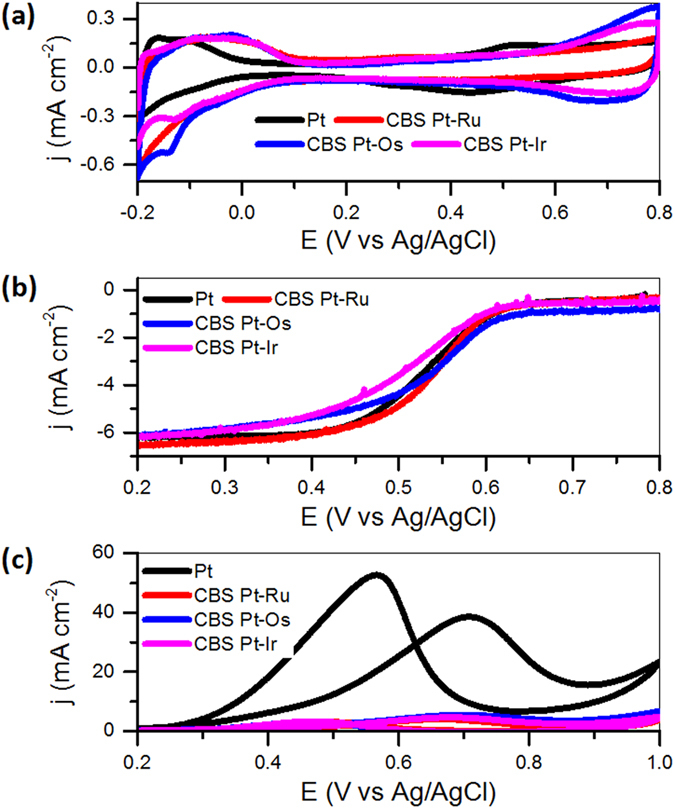
Electrochemical measurements. Cyclic voltammograms of Pt/C, CBS Pt-Ru/C, CBS Pt-Os/C, and CBS Pt-Ir/C catalysts in argon-purged HClO_4_ (0.1 M) at 50 mV s^−1^ (**a**); ORR polarization curves for Pt/C, CBS Pt-Ru/C, CBS Pt-Os/C, and CBS Pt-Ir/C catalysts in an O_2_-saturated HClO_4_ solution (0.1 M) at 20 mV s^−1^ and a rotating speed of 1600 rpm (**b**); Cyclic voltammograms of Pt/C, CBS Pt-Ru/C, CBS Pt-Os/C, and CBS Pt-Ir/C catalysts in argon-purged HClO_4_ (0.1 M) with 1 M methanol at 20 mV s^−1^ (**c**).

The electrochemical active surface areas (ECSAs) of Pt/C, CBS Pt-Ru/C, CBS Pt-Os/C, and CBS Pt-Ir/C catalysts were measured by cyclic voltammetry (Fig. 5a). The ECSAs normalized by the mass of Pt are 30.6 m^2^ g_Pt_^−1^, 26.2 m^2^ g_Pt_^−1^, 25.4 m^2^ g_Pt_^−1^, and 26.8 m^2^ g_Pt_^−1^ for Pt/C, CBS Pt-Ru/C, CBS Pt-Os/C, and CBS Pt-Ir/C, respectively. The presence of residual impurities adsorbed on the surface might be harmful to the active surface areas of the nanoparticles. However, the comparable ECSAs of the Pt seeds and CBS Pt-M nanoparticles suggest that the effect of the porous metal shell (Ru, Os, or Ir) in CBS particles on the ECSAs of the Pt core could be negligible.

Figure 5b shows the ORR polarization curves in the potential range of 0.8 to 0 V for the Pt/C and CBS Pt-M/C catalysts in oxygen-saturated 0.1 M HClO_4_ at room temperature. For the Pt/C, CBS Pt-Ru, CBS Pt-Os, and CBS Pt-Ir catalysts, the half-wave potentials are 529 mV, 532 mV, 535 mV, and 522 mV, respectively. The CBS Pt-M nanoparticles display very comparable activity with that of Pt seed particles for ORR due to the same size of their active metal, suggesting the porous Ru, Os, or Ir shell has negligible effect on the activity of Pt core for ORR.

The activity trend of the Pt/C and CBS Pt-M/C catalysts for MOR is quite different from that for ORR. The low MOR activity of the CBS Pt-M catalysts is a strong contrast to its high ORR activity, as demonstrated by Fig. 5c for the voltammograms. The peak current densities for CBS Pt-Ru/C, CBS Pt-Os/C, and CBS Pt-Ir/C associated with methanol oxidation in the forward scans are 4.2, 5.5, and 4.7 mA cm^−2^, respectively, and are only 10.9%, 14.3%, and 12.2% of the current densities of Pt/C catalysts (38.5 mA cm^−2^). The comparison of current densities shows that the CBS Pt-M nanoparticles have much lower specific activity than that of the Pt seeds. Because of the identical size and morphology of the Pt seeds and Pt core in CBS nanoparticles, the lower catalytic activity of CBS Pt-M nanoparticles could only be attributed to the presence of porous Ru, Os, or Ir shell, which has significant inhibition for the oxidation of methanol on the surface of Pt core in CBS Pt-M nanoparticles.

**Figure 6:**
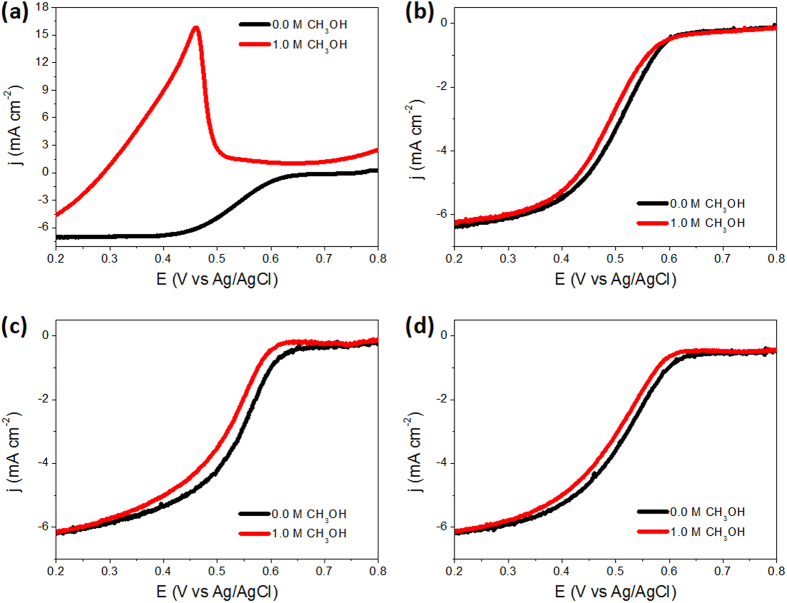
Oxygen reduction with or without methanol. ORR polarization curves for Pt/C (**a**), CBS Pt-Ru/C (**b**), CBS Pt-Os/C (**c**), and CBS Pt-Ir/C catalysts (**d**) in an O_2_-saturated HClO_4_ solution (0.1 M) with or without 1 M methanol at a scan rate of 20 mV s^−1^ and a rotating speed of 1600 rpm.

As further proofs to prove the inhibition of CBS nanostructures on the MOR, Fig. 6 shows the polarization curves of ORR on Pt seeds and CBS Pt-M nanoparticles in the presence of methanol in concentrations as high as 1.0 M in the electrolyte. As displayed by Fig. 6b,c,d, the catalytic reduction of oxygen on the CBS Pt-M nanoparticles was hardly affected. The half-wave potentials of CBS Pt-Ru/C, CBS Pt-Os/C, and CBS Pt-Ir/C catalysts in the presence of methanol can reach 96.2%, 94.6%, and 96.6% of the half-wave potentials of corresponding CBS nanoparticles without methanol, demonstrating the effective inhibition of methanol oxidation on the CBS Pt-M nanoparticles. For comparison, oxygen reduction on the Pt seed particles with and without methanol was also measured (Fig. 6a). The ORR polarization curve in this case was clearly affected in the presence of 1 M methanol: a peak was formed at the potential for methanol oxidation. As we have depicted in early studies^27^,^28^, in the CBS Pt-M catalyst, the catalytically active metal, i.e. Pt, is located in the core region shielded by a porous metal shell. Methanol or oxygen must diffuse through the porous shell of the CBS nanoparticles to access the active Pt core for electrocatalysis to occur. In this case, the larger molecular size of methanol would obstruct its diffusion in CBS nanostructures, rendering the oxidation of methanol on CBS Pt-M a non-competitive event. Chronoamperometries of Pt/C and CBS Pt-M/C catalysts at 0.45 V in oxygen-saturated 0.1 M HClO_4_ solution in the presence of 1 M methanol were used to obtain some indications of the long-term performance of the catalysts in ORR. SI Figure S5 shows that the “steady state” activity of CBS Pt-M nanoparticles is much higher than that of the Pt seeds after more than 5 h, indicating that the Pt catalyst for ORR in the presence of methanol can be stabilized by the porous metal shell. As a typical example, the TEM image of the CBS Pt-Ru nanoparticles after electrochemical measurements was shown by SI Figure S6. As exhibited, no apparent change in size and structure could be observed in comparison with that of the particles before electrochemical measurements (SI Figure S4c), indicating the high structure stability during the electrochemical measurement.

By developing effective strategies to tailor the structures (e.g. the size of Pt core and the porosity of metal shell) of the CBS nanoparticles, one would expect the ORR catalytic activity and methanol-tolerant property of CBS Pt-M nanoparticles could be further enhanced. Although this concept has been advanced and proved in our early studies, the merit of using remarkably inexpensive chemical agent (NaCl) in this work to promote the formation of CBS particles containing a wide spectrum of metal shells should be emphasized, and it might have provided a promising method for creating highly selective electrocatalysts on a large scale for the cathode reaction of DMFCs.

In summary, we have developed a cost-effective approach for the fabrication of bimetallic Pt-M nanoparticles with a cage-bell structure, defined as a movable Pt core enclosed by a metal shell with nano-channels. This approach is based on the removal of the inner Ag shell from Pt-Ag-M nanoparticles with core-shell-shell structure using inexpensive NaCl. In this strategy, core-shell-shell nanoparticles with Pt and Ag residing respectively in the core and inner shell regions were first prepared in an organic solvent, which were then agitated with saturated NaCl solution to eliminate the Ag component from the inner shell region, leading to the formation of CBS Pt-M nanoparticles. The electrochemical measurments demonstrated that the NaCl induced CBS Pt-M nanoparticles supported on carbon support could enhance the methanol-tolarent properties while remain the ORR activity of the Pt seeds by inhibiting the diffusion of methanol in their porous metal shell. In particular, the remarkably inexpensive chemical agent (NaCl) used to promote the formation of CBS particles containing a wide spectrum of metal shells may highlight the merit to produce highly selective electrocatalysts on a large scale for the cathode reaction of DMFCs.

## Methods

### General materials

Platinum(II) acetylacetonate (Pt(acac)_2_, 97%), potassium tetrachloroplatinate(II) (K_2_PtCl_4_, 98%), ruthenium(III) chloride (RuCl_3_, Ru content 45%–55%), osmium(III) chloride (OsCl_3_, 99.9%), and iridium(III) acetylacetonate (Ir(acac)_3_, 97%) from Sigma-Aldrich, oleylamine (95.4%, primary amine) from J&K Scientific, silver nitrate (AgNO_3_, 99%), aqueous HClO_4_ solution (70%, ACS reagent), and Nafion 117 solution (5% in a mixture of lower aliphatic alcohols and water) from Aladdin Reagents, sodium chloride (NaCl, analytical grade) from Xilong Chemical Co., Ltd., acetic acid (C_2_H_4_O_2_, analytical grade), methanol (99%), and toluene (99.5%) from Beijing Chemical Works, and Vulcan XC-72 carbon powders (XC-72C) with BET surface area of 250 m^2^ g^−1^ and average particle size of ca. 40 nm from Cabot Corporation, were used as received. All glassware and Teflon-coated magnetic stir bars were cleaned with *aqua regia*, followed by copious washing with de-ionized water before drying in an oven.

### Synthesis of Pt seed particles

In a typical synthesis of Pt seed particles, 60 mg of Pt(acac)_2_ and 10 mg of AgNO_3_ were added to 20 mL of oleylamine. The small amount of AgNO_3_ was used to facilitate the formation of Pt nanoparticles with regularly spherical shapes. The mixture was then heated and kept at 185 °C for 2 h with stirring under flowing N_2_. After reaction, the Pt seed particles were purified by precipitation with methanol, followed by centrifugation and washing with methanol, and then re-dispersed in 20 mL of toluene. The effect of the additive AgNO_3_ amount, temperature and different Pt precursors on the average size and morphology of Pt seed particles were also investigated.

### Synthesis of core-shell Pt-Ag and core-shell-shell Pt-Ag-M (M = Ru, Os, or Ir) nanoparticles

Successive reduction, also known as the seed-mediated growth method, was used to obtain Pt-Ag nanoparticles with a core-shell structure. In brief, 60 mg of Pt(acac)_2_ and 10 mg of AgNO_3_ were added to 20 mL of oleylamine. The mixture was heated to and maintained at 185 °C for 2 h under flowing N_2_ and stirring to prepare the Pt seeds. Then the temperature of the reaction mixture was lowered to 100 °C, at which 90 mg of AgNO_3_ was added swiftly and the reaction mixture was maintained at 100 °C under flowing N_2_ for another 3 h for the growth of Ag on existing Pt seeds.

Subsequently, for the synthesis of core-shell-shell Pt-Ag-M (M = Ru, Os, or Ir) nanoparticles, 70 mg of RuCl_3_, 53 mg of OsCl_3_, or 88 mg of Ir(acac)_3_ was added swiftly, followed by heating and keeping the reaction mixture at 230 °C for 1.5 h under flowing N_2_ for the reduction of the noble metal precursors in the presence of previously formed Pt-Ag nanoparticles. After the reaction, these core-shell-shell Pt-Ag-M nanoparticles were purified by precipitation with methanol, centrifugation, washing with methanol, and re-dispersed in 20 mL of toluene.

### Synthesis of cage-bell structured Pt-M (CBS Pt-M, M = Ru, Os, or Ir) nanoparticles

To remove the inner Ag shell from the core-shell-shell Pt-Ag-M nanoparticles for the formation of CBS Pt-M nanoparticles, the core-shell-shell Pt-Ag-M nanoparticle solution was mixed with saturated aqueous solution of NaCl, and the mixture was aged for 12 h under vigorous stirring at room temperature. Then the upper toluene phase containing CBS Pt-M nanoparticles was collected after complete separation of the two phases.

### Particle characterizations

Transmission electron microscopy (TEM) and high resolution TEM (HRTEM) were performed on the JEOL JEM-2100F electron microscope operating at 200 kV. For the TEM measurements, a drop of the nanoparticle solution was dispensed onto a 3-mm carbon-coated copper grid. Excessive solution was removed by an absorbent paper, and the sample was dried under vacuum at room temperature. An energy dispersive X-ray spectroscopy (EDX) analyzer attached to the TEM was used to analyze the chemical compositions of the synthesized nanoparticles. UV-visible spectra of the core-shell and CBS particle solutions were collected on a Hitachi U-3900 spectrophotometer. X-ray photoelectron spectroscopy (XPS) was conducted on a VG ESCALAB MKII spectrometer. Sample preparation for XPS analysis began with concentrating 5 mL of the toluene solution of the metal nanoparticles to 0.5 mL using flowing N_2_. 10 mL of methanol was then added to precipitate the metal nanoparticles. The nanoparticles were then recovered by centrifugation and washed with methanol several times to remove non-specifically bound oleylamine. The nanoparticles were then dried at room temperature in vacuum.

### Electrochemical measurements

Electrochemical measurements were carried out in a standard three-electrode cell connected to a Bio-logic VMP3 (with EC-lab software version 9.56) potentiostat. A leak-free Ag/AgCl (saturated with KCl) electrode was used as the reference electrode. The counter electrode was a platinum mesh (1 × 1 cm^2^) attached to a platinum wire.

For the loading of the Pt seed particles and CBS Pt-M nanoparticles on Vulcan XC-72 carbon support, a calculated amount of carbon powder was added to the toluene solution of Pt seeds or CBS Pt-M nanoparticles. After stirring the mixture for 24 h, the Pt/C or CBS Pt-M/C catalysts (20 wt% Pt on carbon support) were collected by centrifugation, washed thrice with methanol, and then dried at room temperature in vacuum.

The working electrode was a thin layer of Nafion-impregnated catalyst cast on a vitreous carbon disk. This electrode was prepared by ultrasonically dispersing 10 mg of the Pt/C or CBS Pt-M/C catalysts in 10 mL of water containing 4 mL of ethanol and 0.1 mL of Nafion solution. A calculated volume of the ink was dispensed onto the 5 mm glassy carbon disk electrode to produce a nominal catalyst loading of 20 μg cm^−2^ (Pt basis). The carbon electrode was then dried in a stream of warm air at 70 °C for 1 h.

The room temperature cyclic voltammograms of Pt/C and CBS Pt-M/C in argon-purged HClO_4_ (0.1 M) were recorded between −0.2 V and 0.8 V at 50 mV s^−1^ and used to determine the electrochemically active surface areas (ECSAs) of Pt. The performance of Pt seeds and CBS Pt-Ru nanoparticles in room-temperature MOR was measured by cyclic voltammetry. For these measurements the potential window of 0.2 V to 1 V was scanned at 20 mV·s^−1^ until a stable response was obtained. The electrolyte was methanol (1 M) in perchloric acid (0.1 M).

The performance of Pt seeds and CBS Pt-M nanoparticles in room temperature ORR was also evaluated in 0.1 M HClO_4_ electrolyte solution using a glass carbon rotating disk electrode (RDE) at a rotation rate of 1600 rpm. A solution of 1 M methanol in 0.1 M HClO_4_ was used for testing the methanol tolerance of the CBS Pt-M nanoparticles. Negative-going linear sweep voltammograms were recorded from 0.8 V to 0.2 V at 20 mV·s^−1^ at room temperature in the presence of bubbling ultra-pure oxygen to maintain a saturated oxygen atmosphere near the working electrode.

## Additional Information

**How to cite this article**: Feng, Y. *et al.* Enhancing the methanol tolerance of platinum nanoparticles for the cathode reaction of direct methanol fuel cells through a geometric design. *Sci. Rep.* **5**, 16219; doi: 10.1038/srep16219 (2015).

## Supplementary Material

Supplementary Information
