# Supplementary material for: Enhancing the methanol tolerance of platinum nanoparticles for the cathode reaction of direct methanol fuel cells through a geometric design
Source: Sci Rep. 2015 Nov 18;5:16219. doi: 10.1038/srep16219 (PMC4649704; doi:10.1038/srep16219)
Supplement: Supplementary Information [file srep16219-s1.doc]

Supplementary Information

**Enhancing the methanol tolerance of platinum nanoparticles for the cathode reaction of direct methanol fuel cells through a geometric design**

Yan Feng^1,2^, Feng Ye^1^, Hui Liu ^1,3^,* & Jun Yang ^1,3^,*

^1^State Key Laboratory of Multiphase Complex Systems, Institute of Process Engineering, Chinese Academy of Sciences, Beijing, China 100190. Fax: 86-10-8254 4915; Tel: 86-10-8254 4915; E-mail: liuhui@ipe.ac.cn (HL); [jyang@ipe.ac.cn](mailto:jyang@mail.ipe.ac.cn) (JY)

^2^University of Chinese Academy of Sciences, No. 19A Yuquan Road, Beijing, China 100190

^3^Center for Mesoscience, Institute of Process Engineering, Chinese Academy of Sciences, Beijing, 100190, China

Financial support from the National Natural Science Foundation of China (Nos.: 21173226, 21376247, and 21476246), and Center for Mesoscience, Institute of Process Engineering, Chinese Academy of Sciences (COM2015A001) is gratefully acknowledged.


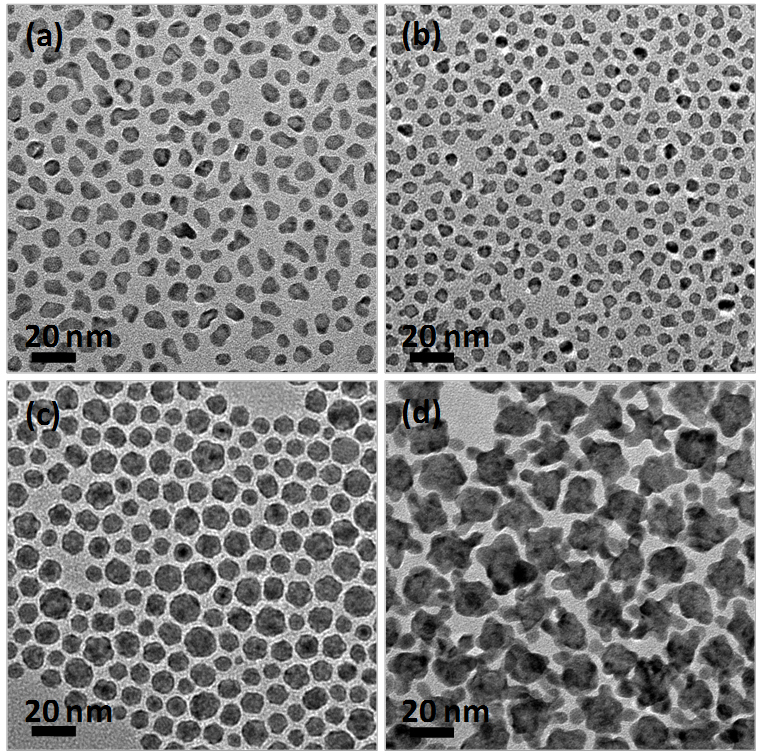


**Figure S1.** **Pt seed particles using other Pt precursors.** TEM images (a,b,c) of the Pt seed particles synthesized at 185°C using Pt(acac)_2_ as Pt precursors with the additive AgNO_3_ of 5 mg (a), 15 mg (b), and 20 mg (c), respectively; TEM image (d) of the Pt seed particles synthesized at 185°C using K_2_PtCl_4_ as Pt precursors with the additive AgNO_3_ of 10 mg.


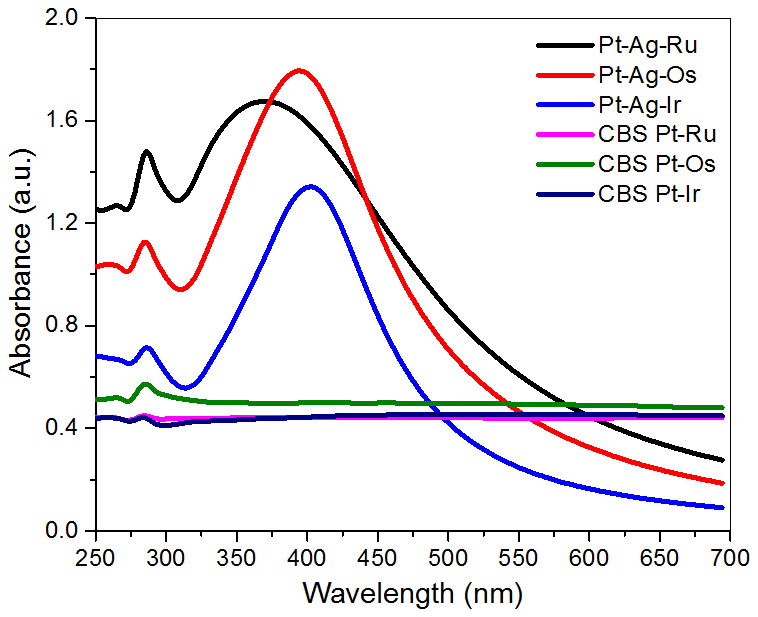


**Figure S2.** **UV-visible characterizations.** UV-visible spectra of core-shell-shell Pt-Ag-M nanoparticles before and after treatment with saturated aqueous NaCl solution.


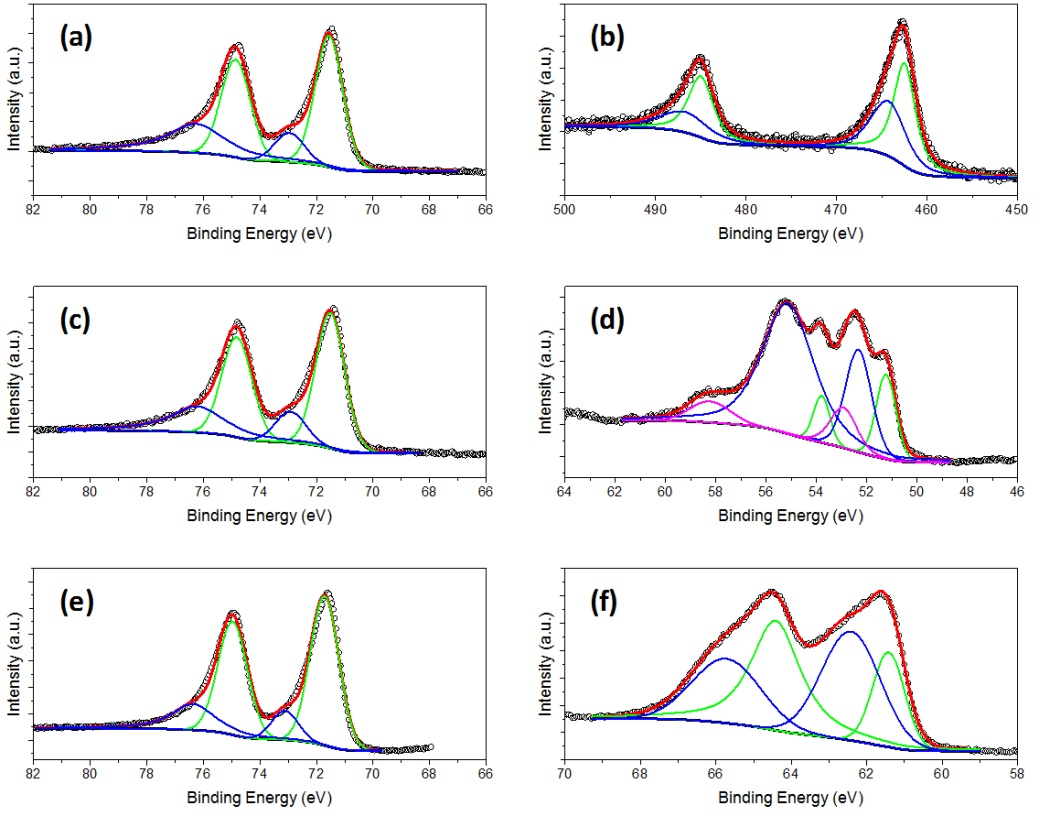


**Figure S3.** **XPS characterizations.** XPS spectra of Pt 4f (a,c,e), Ru 3p (b), Os 4f (d), and Ir 4f (f) in CBS Pt-Ru (a,b), CBS Pt-Os (c,d), and CBS Pt-Ir nanoparticles (e,f), respectively.


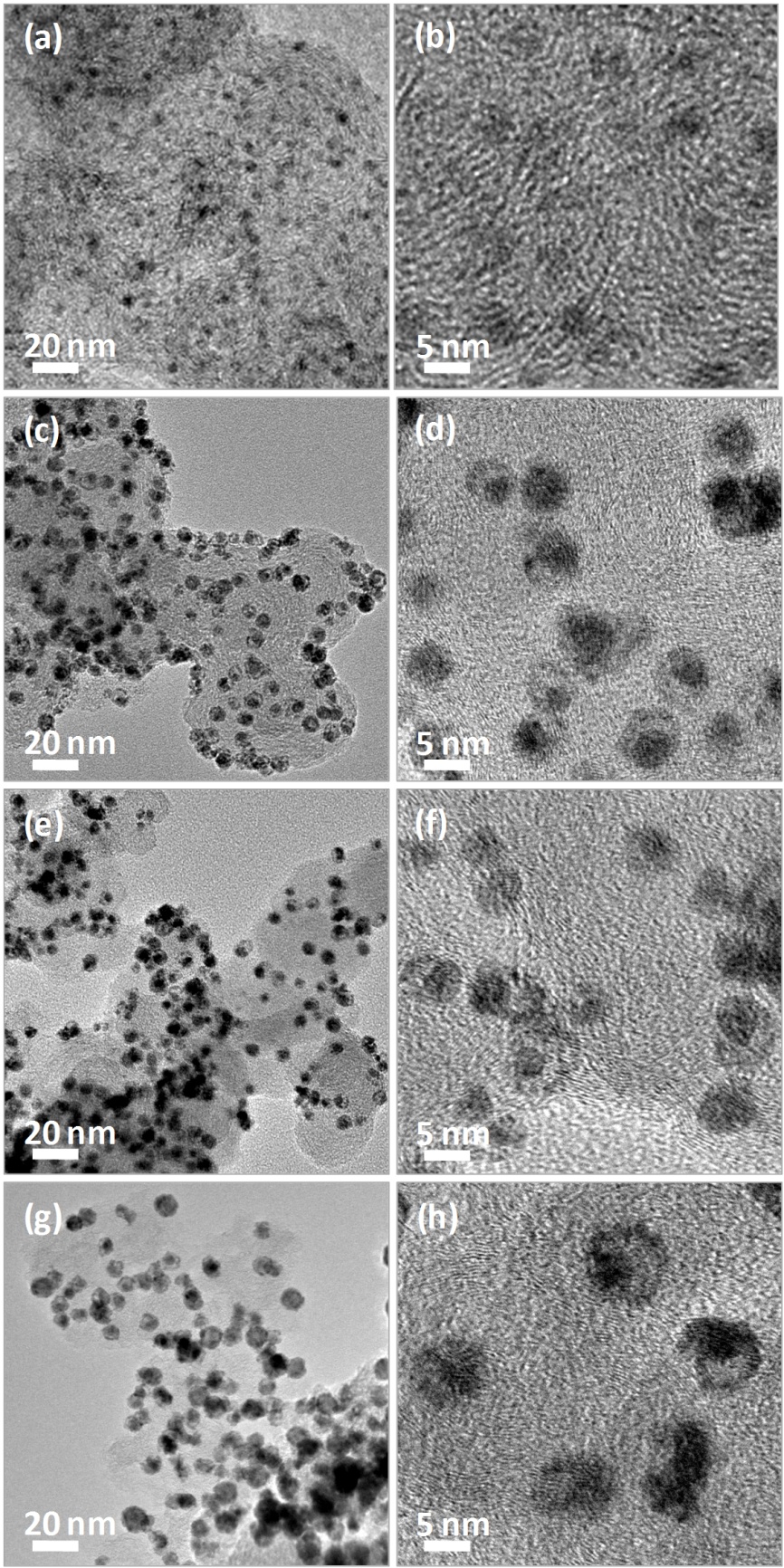


**Figure S4.** **Carbon-supported CBS Pt-M nanoparticles.** TEM images (a,c,e,g) and HRTEM image (b,d,f,h) of Pt seeds (a,b), CBS Pt-Ru (c,d), CBS Pt-Os (e,f), and CBS Pt-Ir (g,h) nanoparticles loaded on carbon supports.


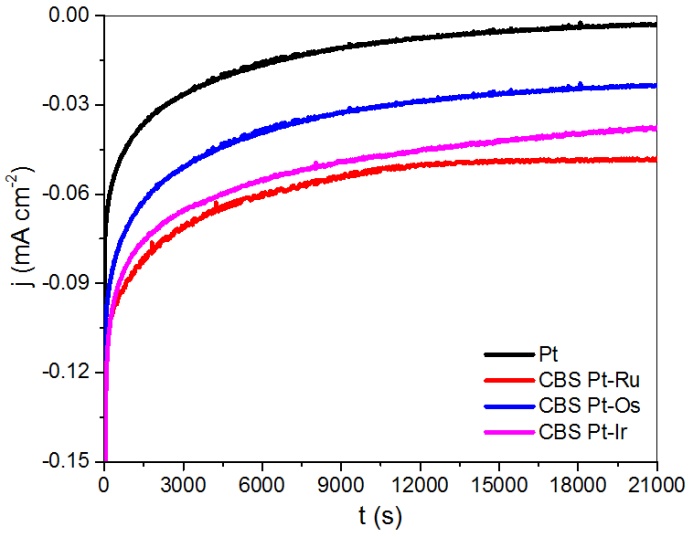


**Figure S5.** **Long-term performance of CBS Pt-M nanoparticles for oxygen reduction.** Chronoamperograms at 0.45 V of CBS Pt-Ru, CBS Pt-Os, and CBS Pt-Ir nanoparticles in O_2_ saturated 0.1 M HClO_4_ electrolyte at a scan rate of 20 mV s^-1^ and a rotating rate of 1600 rpm.

**Figure S6. Carbon-supported CBS Pt-Ru nanoparticles after electrochemical measurements.** TEM images of carbon-supported CBS Pt-Ru nanoparticles after electrochemical measurement.
